# Use of ‘acute behavioural disturbance’ in mental health records: differences over time and by ethnicity in a London NHS mental health Trust

**DOI:** 10.1192/bjo.2023.528

**Published:** 2023-07-24

**Authors:** Catherine Polling, Preety Das, Kevin Ariyo, Natalie Creary, Shubulade Smith

**Affiliations:** Department of Psychological Medicine, Institute of Psychiatry, Psychology and Neuroscience, King's College London, UK; and Psychological Medicine and Integrated Care, South London and Maudsley NHS Foundation Trust, UK; Department of Psychological Medicine, Institute of Psychiatry, Psychology and Neuroscience, King's College London, UK; Black Thrive Lambeth, UK; Behavioural and Developmental Disorders Directorate, South London and Maudsley NHS Foundation Trust, UK; and Department of Forensic and Neurodevelopmental Science, Institute of Psychiatry, Psychology and Neuroscience, King's College London, UK

**Keywords:** Epidemiology, stigma and discrimination, risk assessment, emergency presentations, liaison psychiatry

## Abstract

**Background:**

Acute behavioural disturbance (ABD) is a controversial descriptor for presentations of severe agitation, aggression and physiological compromise.

**Aims:**

To characterise the use of ABD-related terms in the electronic record of a large UK provider of mental health services during 2006–2021.

**Method:**

The free text of all records relating to patient contacts with acute assessment mental health teams during 2006–2021 were searched for references to ABD. Identified text was coded for context of use and presence of clinical features of ABD described in the literature. Poisson regression was used to analyse differences in rates of use over time and between demographic groups.

**Results:**

Mentions of ABD increased by an average of 1.12 (95% confidence interval (CI), 1.08–1.17) per year, with the greatest increase from 2019 to 2021. Black people were more than twice as likely as White people to have reference to ABD included in their assessments (rate: 2.4/1000 (95% CI 1.8–3.1) in Black people compared with 1.0/1000 (95% CI 0.8–1.3) in White people). The clinical characteristics in notes describing a current presentation of ABD rarely corresponded to those included in UK medical guidelines on ABD.

**Conclusions:**

The term ABD in mental health notes appears to often, but not exclusively, be a synonym for severe agitation and conveys little meaning beyond this. However, the term's connection to a literature emphasising the high risk of physical health collapse and need for urgent treatment means that its disproportionate use in Black people may contribute to existing racial inequalities in the use of coercive measures during crisis presentations.

‘Acute behavioural disturbance’ (ABD) is a phrase used largely by non-psychiatric emergency staff to describe a clinical presentation of highly agitated, disturbed and often aggressive behaviour and clinical signs of physiological compromise.^1^ It differs from the longstanding use by psychiatrists of terms such as ‘acutely behaviourally disturbed’ as broad descriptors, by being framed as a specific clinical entity described by a set of signs and symptoms and linked to poor physical health outcomes.^2^ As such, ABD has featured in the Royal College of Emergency Medicine guidelines^2^ and was included in the Maudsley Prescribing Guidelines for the first time in its most recent edition.^3^ These refer to ABD as synonymous with the term ‘excited delirium’, more commonly used in North America, and the clinical features attributed to it draw on the excited delirium literature.^2^ Both terms are contested within medicine. Neither term is included in either the World Health Organization's ICD-10^4^ or the American Psychiatric Association's DSM-5,^5^ and neither has a clear, agreed definition.^6,7^ Authors in both the UK and USA have highlighted concerns about its use as the explanatory cause of death in situations where people have died under restraint, and in particular, its greater use in Black men.^8–10^ However, others have argued that it merits recognition as a ‘potentially life-threatening syndrome’ linked to rapid deterioration and death, which is useful for emergency responders to be able to recognise.^1^ In response to this ongoing controversy, the Royal College of Psychiatrists set up an Expert Reference Group to produce a position paper on the issue. The recently published Position Statement from this group has noted the absence of reliable data on the frequency with which the term ABD is being used, the uncertainty around definitions when it is used and the history of disproportionate use of the term in people from racialised groups, especially Black men.^11^

## Aims

This study aimed to characterise the use of the term ABD and related terms in the electronic record of a large provider of mental health services between 2006 and 2021. It aimed to answer the questions: (a) is the frequency of these terms in notes increasing over time?; (b) are the terms more likely to be used about people from racialised minorities than White people? and (c) when the terms are used, are the authors referring to the group of symptoms described in UK clinical guidelines relating to ABD?

## Method

### Data

Data were accessed via the Clinical Records Interactive Search system (CRIS), a case register created from the anonymised electronic patient record of the South London and Maudsley NHS Foundation Trust (SLaM).^12^ CRIS contains the entire contents of the electronic record from 2006 onward, including free-text entries made by mental health professionals. The SLaM provides all secondary mental healthcare for the four London boroughs of Lambeth, Southwark, Lewisham and Croydon, with a population of 1.2 million that is diverse in terms of ethnicity and socioeconomic status.

We searched the free text of all records relating to patient contacts with mental health teams that do acute, unplanned assessments, which we defined as general hospital liaison (emergency department and in-patient), crisis/home treatment teams and health-based places of safety, in patients of all ages. We identified records that contained the phrases ‘acute behavio(u)ral disturbance’, acute behavio(u)ral disorder’, ‘ABD’ and ‘excited delirium’. Where any of these phrases occurred, we extracted the full free-text entry and structured data relating to the date, time and location of the contact; the age, gender and ethnicity of the patient and professional designation of the author of the entry.

### Ethics

Research using data within CRIS is covered by a database approval from Oxford Research Ethics Committee C (approval number 08/H0606/71+5), this project was approved by the CRIS Oversight Committee (project number 22-029). In line with this approval, statistical cell sizes below ten are not reported in this paper and free-text extracts are limited to brief exerts that do not include any patient-identifiable information.

### Analysis

The identified free text was read by one of two coders (C.P. and P.D.). Initially, a randomly selected set of 20 entries was coded by both coders, agreement between the coders was checked and coding rules were amended where ambiguity had been identified. In a first round of coding, the context in which ABD and related phrases were being used was identified as referring to (a) reason for presentation and/or author's current impression following assessment, (b) past history only, (c) plan for future possibility only or (d) absence noted.

To allow comparisons of the frequency of ABD and related phrases in notes over time, rates were calculated per 100 000 free-text entries relating to any contact with acute assessing teams each year, to account for an increasing number of entries in the electronic record over time. Change in rates over time were quantified with Poisson regression to give rate ratios for change per 1 year. For comparisons by ethnicity, rates were calculated per 1000 individuals within CRIS who had at least one entry made by an acute assessment team.

There are no clinical guidelines referring to ABD produced by the Royal College of Psychiatrists. Instead, relevant clinical signs associated with ABD were identified from guidelines produced by other medical royal colleges: the Royal College of Emergency Medicine guidelines issued in 2016^13^ and 2022;^2^ the Royal College of Physicians Faculty of Forensic & Legal Medicine guidelines in 2019^14^ and the Maudsley Prescribing Guidelines,^3^ a widely used reference in UK psychiatry.

For those records where current ABD was being documented, notes were coded for whether either presence or absence of the identified clinical signs had been documented. In addition to these clinical signs, entries relating to a current ABD presentation were also coded according to the most likely aetiology identified by the clinician making the entry. Based on discussions with community stakeholders, notes were also coded according to whether there was any mention of police and/or ambulance involvement in the individual's presentation for assessment, and whether any friend or family member was present for the assessment.

All analyses were performed in R for Windows version 3.4.2 (R Foundation for Statistical Computing, Vienna, Austria; see https://www.R-project.org).

## Results

### Characteristics of the sample

There were 402 free-text entries (‘documents’) made by acute assessment teams between 2006 and 2021 that contained ABD or the related terms searched for (‘ABD terms’). These related to 318 assessments of 307 individuals, the majority of whom had only one assessment in the time period in which ABD terms were used (296/307, 96.4%).

In 94% (300/318) of assessments, the phrase used was ABD. The phrase ‘excited delirium’ was used in only four assessments. Most assessments where ABD terms were documented were made by doctors (261/318, 82.1%) and were made in general hospital settings (270/318, 84.9%), with most of the rest occurring in health-based places of safety 12.3% (39/318). These were all doctors working in mental health teams, and will include both doctors of all grades working in liaison psychiatry teams and psychiatric trainees working on-call out of hours.

Table 1 shows the characteristics of patients with ABD terms documented in their assessments, stratified by the context in which ABD phrases were being used. In 61% (194/318) of assessments, ABD phrases referred to the current presentation, either within the description of patient's presenting circumstances (e.g. ‘sudden onset of acute behavioural disturbance this morning’) or in the documented clinical impression (e.g. ‘likely presentation of acute behavioural disturbance in the context of polysubstance and alcohol misuse’). A further 18% were references to the possibility of future behavioural disturbance within management plans (e.g. ‘use lorazepam/promethazine in the event of acute behavioural disturbance’). The remainder either documented a history of previous ABD (32/318, 10%) (e.g. in a description of a previous episode, ‘required 1:1 input due to acute behavioural disturbance’) or the absence of ABD in the current presentation (36/318, 11%) (e.g. ‘did not display any features of acute behavioural disturbance’).

**Table 1:** Description of sample by acute behavioural disturbance context

	Absence, *n* (%)	Current, *n* (%)	Past history, *n* (%)	Future plan, *n* (%)
Total	36	194	32	56
Age				
Child and adolescent	<10 (8.3%)	17 (8.8%)	<10 (6.2%)	<10 (5.4%)
Working age	23 (63.9%)	155 (79.9%)	27 (84.4%)	44 (78.6%)
Old age	10 (27.8%)	22 (11.3%)	<10 (9.4%)	<10 (16.1%)
Gender				
Female	18 (50%)	82 (42.3%)	12 (37.5%)	31 (56.4%)
Male	18 (50%)	112 (57.7%)	20 (62.5%)	24 (43.6%)
Ethnicity				
White	17 (54.8%)	74 (47.1%)	13 (46.4%)	31 (63.3%)
White British	15 (48.4%)	56 (35.7%)	<10 (32.1%)	25 (51%)
White Irish	<10 (0%)	<10 (3.2%)	<10 (3.6%)	<10 (2%)
White other	<10 (6.4%)	13 (8.3%)	<10 (10.7%)	<10 (10.2%)
Mixed	<10 (6.4%)	<10 (1.3%)	<10 (0%)	<10 (2%)
Asian	<10 (9.7%)	<10 (4.5%)	<10 (14.3%)	<10 (4.1%)
Black	<10 (22.6%)	57 (36.3%)	<10 (32.1%)	12 (24.5%)
Black Caribbean	<10 (9.7%)	13 (8.3%)	<10 (0%)	<10 (8.2%)
Black African	<10 (6.4%)	19 (12.1%)	<10 (10.7%)	<10 (10.2%)
Black other	<10 (6.4%)	25 (15.9%)	<10 (21.4%)	<10 (6.1%)
Other	<10 (6.4%)	17 (10.8%)	<10 (7.1%)	<10 (6.1%)

The individuals documented as having current ABD ranged in age from below 10 to over 90 years, but 80% were of working age, with a median age of 33 (interquartile range: 25–47) years. There were more men than women documented as currently (58%) or previously (63%) having ABD. There was police involvement in 37% (72/194) of the presentations where current ABD was documented: this included all of the assessments in a health-based place of safety or police station, as well as 46% (40/88) of the emergency department assessments. Fewer than a quarter of the assessments (45/194, 23%) document the presence of a friend or family member accompanying the person assessed.

### Changes over time

**Fig. 1:**
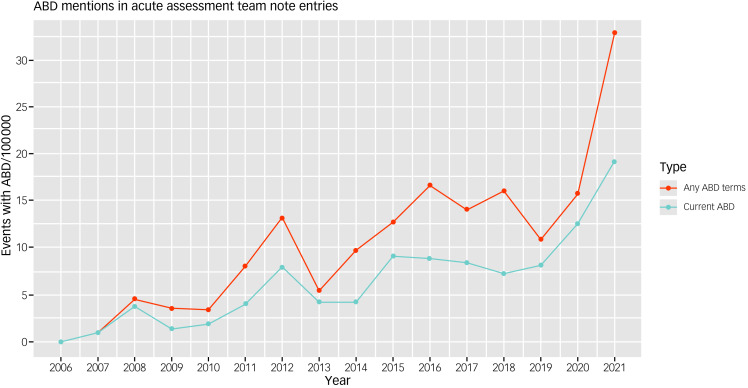
Change in ABD use over time. ABD, acute behavioural disturbance.

Figure 1 shows the change in the rate of occurrence of ABD terms in any context and assessments documenting current ABD, in acute assessment documents between 2006 and 2021. Both increase over the study period, with the greatest increase between 2019 and 2021. Poisson regression calculated an average rate increase of 1.12 (95% CI 1.08–1.17) per year in assessments documenting current ABD over the study period, representing a 5.4-times increase in the rate over the 15 years of the study period. Supplementary Table 1 available at https://doi.org/10.1192/bjo.2023.528 shows counts and rates per year for all ABD terms and current ABD.

### Ethnicity

Information on ethnicity was available for 83% (254/307) of individuals with any mention of ABD terms and 81% (154/191) of those assessed at least once as currently having ABD. Table 2 shows that the people of Black ethnicity are overrepresented among those with reference to current ABD in their notes compared with their proportion in the population served by the mental health Trust (37.0% of those with current ABD *v*. 24.7% of the population), with the largest disparity in the Black other group (16.2 *v*. 4.2%). Conversely the White, Asian and Mixed groups are underrepresented compared with their proportions in the underlying population.

**Table 2:** Frequency of use of acute behavioural disturbance, by ethnic group

Ethnic group	Individuals with current ABD documented, *n* (%)	Population at 2011 Census (1000s), *n* (%)	Individuals with entries in acute settings, *n*	Current ABD/1000 individuals (95% CI)
**White**	**73 (47.4%)**	**677.3 (55%)**	**72 051**	**1.0 (0.8–1.3)**
White British	55 (35.7%)	519 (42.2%)	59 318	0.9 (0.7–1.2)
White Irish	<10 (3.2%)	24.3 (2%)	2542	2.0 (0.6–4.6)
White other	13 (8.4%)	134 (10.9%)	10 191	1.3 (0.7–2.2)
**Mixed**	**<10 (1.3%)**	**85.3 (6.9%)**	**3333**	**0.6 (0.1–2.2)**
**Asian**	**<10 (3.9%)**	**110.6 (9%)**	**6993**	**0.9 (0.3–1.9)**
**Black**	**57 (37%)**	**304.3 (24.7%)**	**24 148**	**2.4 (1.8–3.1)**
Black African	13 (8.4%)	109 (8.9%)	8331	2.2 (1.2–3.7)
Black Caribbean	19 (12.3%)	143.6 (11.7%)	6015	2.3 (1.4–3.6)
Black other	25 (16.2%)	51.6 (4.2%)	9802	2.6 (1.7–3.8)
**Other ethnic group**	**16 (10.4%)**	**53.3 (4.3%)**	**8193**	**2.0 (1.1–3.2)**

ABD, acute behavioural disturbance. Ethnic groups in bold are ONS higher-level groups.

When the rate of terms describing current ABD per 1000 individuals assessed in acute medical settings is considered, rates are highest in the Black group, at 2.4/1000 individuals assessed (95% CI 1.8–3.1) compared with 1.0/1000 people assessed (95% CI 0.8–1.3) for the White group. The similar, higher rates are seen in all three of the more specific Black ethnic groups.

### Documentation of clinical features and likely aetiology

Six groups of symptoms were described in all of the Royal College of Emergency Medicine guidelines,^13,14^ the Royal College of Physicians Faculty of Forensic & Legal Medicine guidelines^2^ and the Maudsley Prescribing Guidelines:^3^ violent behaviour/extreme agitation or aggression, increased pain tolerance, constant activity, rapid breathing, lack of fatigue and hot to touch/measured raised temperature/profuse sweating. A further two feature in the 2022 Royal College of Emergency Medicine guidelines:^2^ naked/inappropriately clothed and excessive strength/continued struggle despite restraint. Two further symptoms appear in the older Royal College of Emergency Medicine guidelines: unresponsive to other's presence and glass attraction/destruction.

Table 3 shows the frequency with which each of the identified clinical signs was documented in entries related to current presentations with ABD. Violent behaviour/extreme agitation/aggression was documented in 64% of entries (124/194); however, all of the other clinical signs of ABD referred to in UK guidelines were rarely mentioned. Only 23/194 (12%) of entries contain references to the presence of any other sign. When reading the text, it was noted that the phrase ABD was most often being used as a synonym for agitation.

**Table 3:** Frequency of documentation of relevant clinical signs

Clinical sign	Documented as present, *n* (%)
Violent behaviour/extreme agitation/aggression	124 (63.9%)
Increased pain tolerance	0 (0.0%)
Constant activity	1 (0.5%)
Rapid breathing	1 (0.5%)
Lack of fatigue	0 (0.0%)
Tactile hyperthermia/raised temperature/profuse sweating	8 (4.1%)
Naked/inappropriately clothed	7 (3.6%)
Excessive strength/continued struggle despite restraint	1 (0.5%)
Not responsive to others’ presence	4 (2.1%)
Glass attraction/destruction	4 (2.1%)
Any	130 (67.0%)
Any except agitation	23 (11.9%)

The likely aetiologies documented for the presentations are shown in Table 4. A quarter (48/194) of presentations with references to current ABD were attributed to drug use with or without alcohol, and 22% (42/194) to an underlying psychotic or manic illness. Other likely aetiologies included delirium attributed to acute medical illness (11%); alcohol intoxication alone (10%); organic neuropsychiatric causes including seizures, head injury and encephalitis (9%); and behaviours associated with dementia, intellectual disability and autism spectrum disorders (8%).

**Table 4:** Likely aetiology identified in assessments where current presentation was recorded as acute behavioural disturbance, 2006–2021

Likely aetiology	Assessments, *n* (%)
Drug use with or without alcohol	48 (24.7%)
Psychosis/mania	42 (21.6%)
Delirium (medically unwell)	21 (10.8%)
Alcohol intoxication	20 (10.3%)
Organic neuropsychiatric cause	17 (8.8%)
Behaviours associated with intellectual disability/autism spectrum disorders	10 (5.2%)
Dementia	6 (3.1%)
Other	18 (9.3%)
None documented	12 (6.2%)

## Discussion

We found that use of ABD in mental health patient records in South London has increased substantially during the period 2006–2021, with the largest increase occurring in the final 2 years of the study period. The pattern seen was similar for both all mentions of the phrase and instances where the professional writing the record was noting that the patient currently presented with ABD. In contrast, the term ‘excited delirium’ was almost never used at any point in this time period. Therefore, the increasing use of the term ABD does not represent a shift from one terminology to another, but increased naming of a concept that did not previously feature in mental health records.

The recent Royal College of Psychiatrists’ Position Statement on ‘Acute Behavioural Disturbance and Excited Delirium’ notes that the term ABD is more often used by emergency responders and in emergency departments than by psychiatrists.^11^ However, this sample suggests that this terminology is increasingly being used in mental health records by psychiatrists and psychiatric liaison nurses working in acute medical settings such as emergency departments. This increase may reflect an increasing use of the term in medical literature resulting in a greater awareness of the phrase by psychiatrists. Although the publication of the 2021 edition of the Maudsley Prescribing Guidelines containing an entry on ABD^3^ comes at the end of the study period, ABD's inclusion in the guideline likely reflects growing awareness of ABD as a discreet concept relevant to psychiatrists.

The analysis of the clinical symptoms recorded in mental health notes when ABD was mentioned suggests that the term is being used non-specifically to describe severe agitation; indeed, in the large majority of cases this was the only symptom of ABD that was recorded. ABD was rarely being used in a way that referred to the cluster of symptoms described in the UK clinical guidelines we reviewed,^2,3,13,14^ or the research that underpins them.^15^ In particular, the professionals making the records only referenced signs relating to physiological dysregulation or deterioration (such as raised temperature, sweating and rapid breathing) in a tiny minority (<5%) of the cases they were describing as ABD. This study uses data recorded in mental health records, which will not systematically capture physical health diagnoses or treatment received. Hence, it is not possible to address whether those documented as having ABD were at increased risk of physiologically adverse outcomes. Exploring this is an important question for future research. However, the documentation from mental health professionals used in this study suggests that they were rarely associating ABD with the physiological compromise that is supposed to be a key feature. In a third of cases, the records that described a current presentation with ABD did not include a description of severe agitation, violence or aggression. In these cases, notes referred to a wide range of behaviours perceived as abnormal by the documenting mental health professional, but which do not fit with any of the classic symptoms attributed to ABD.

Psychiatrists have long used terms such as acutely behaviourally disturbed, or ‘acute disturbance’, as described in the definition offered by the British Association of Psychopharmacology and National Association of Psychiatric Intensive Care and Low Secure Units joint guidance,^16^ which refers to ‘agitation and distress… that may or may not lead to aggression or violence’. This study's findings raise questions as to what mental health professionals working in acute medical settings and health-based places of safety are referring to when they use the phrase ABD, and how far they are intending to link the presentations they are describing to the concept of ABD in the literature. In its Position Statement,^11^ the Royal College of Psychiatrists discusses the distinction between the broad descriptor ‘acutely disturbed behaviour’ more traditionally used by psychiatrists and ABD ‘which often appears to be a distinct category which is necessarily associated with a significant risk of health emergency’. One possibility is that in more recent times, ‘acute behavioural disorder’ has become something of a stock phrase within mental health records and is assumed to be a synonym for agitation, or even for some professionals, any disordered behaviour. In this case, it carries little clinical diagnostic utility.

However, noting that in reality the phrase often carries little meaning does not mean that its increasing use is not without risk. Much of the criticism that the term ABD has attracted has been because of its use as a justification for both the use and adverse consequences of coercive treatment, on the basis that ABD represents a medical emergency associated with a significant increase in the risk of death.^11,17^ Using the language of ABD, especially in acute medical settings where psychiatrists are communicating with emergency responders and other medical specialities who may have a different understanding of the term, risks widening the group of patients where rapid escalation to more coercive measures such as restraint and use of ketamine^1^ may be seen as warranted.

This is of particular concern given the evidence from this data-set that the use of the term ABD is racialised. Black people were overrepresented among those whose notes contain a mention of current ABD compared with the proportion of people in the population served by the SLaM who are Black, with the largest disparity seen in the Black other group. People of White, Asian and Mixed ethnicities were underrepresented. This is a crude comparison as it does not take into account different age structures and levels of mental ill health in different ethnic groups. However, when rates of documented ABD among those who had at least one contact with an acute assessment team were considered, the same pattern was seen: rates of documented ABD per 1000 individuals assessed in the Black group were more than twice that in the White group. This suggests that the overrepresentation of Black people being documented as having ABD is not simply a result of the known overrepresentation of Black people in crisis presentations:^18^ among those presenting to acute settings, Black people are more likely to be described as having ABD. Despite the fact that when looking at overrepresentation compared with the underlying population there was no disparity for the Black African group and only a small disparity for the Black Caribbean group, when rates per 1000 assessed were considered the disparity became clear: all of the Black ethnic groups had rates more than twice that of the White group.

These findings are in keeping with the research and experiences from the UK and USA reviewed in the Royal College of Psychiatrists’ Position Statement,^11^ which highlights the way that the inclusion of clinical features such as ‘increased pain tolerance’ and ‘excessive strength’ echo racist stereotypes of Black people, particularly men,^8,10^ and questions how the ‘typical case’ of ABD has come to be thought of as a Black man in his 30s, without any critical reflection on how race or gender might predispose an individual's presentation to be perceived in these terms.^11^

### Strengths and limitations

Previous reviews have noted the limited amount of research available on ABD, noting that the nature of an emergency presentation makes it difficult to research.^17^ The use of routine clinical record data creates the opportunity to describe practice over a long study period, capturing data from patient interactions that would be extremely difficult to include in a research study. The data-set also provided denominator data about the number of acute assessments being made overall, allowing more meaningful comparisons of differences by ethnic group and changes over time. However, the nature of the data used means it reflects mental health professionals’ experience of ABD, which may not reflect that of the acute medical responders who have been the main users of the term ABD. Furthermore, only patients who had some contact with mental health professionals will have been included in the sample. Although the acute medical settings included in the study all had a 24 h liaison psychiatry presence throughout the study period, not all presentations with presumed ABD will have necessarily been referred to them.

The use of CRIS allowed us to search the free text of tens of thousands of mental health records for acute presentations, allowing ABD to be identified despite not being a diagnosis that would appear in the coding of routine administrative data. Access to the text of assessments also allowed us to assess the context in which references to ABD were being made. CRIS data covers a single NHS mental health Trust, albeit spread across four general hospitals and other locations. The population served by the Trust is diverse in terms of socioeconomic position and ethnicity, allowing rates for specific ethnic groups to be examined. However, the patterns may reflect influences specific to the Trust, such as habitual practice within certain teams or the effect of Trust-specific training, and so may not be generalisable to other areas.

In conclusion, the use of the phrase ABD in mental health assessments made in acute medical settings increased substantially between 2006 and 2021. People of all Black ethnicities were more than twice as likely as White people to have reference to ABD included in their assessments. However, the clinical characteristics described in notes recording a current presentation of ABD rarely corresponded to those included in UK medical guidelines on ABD. The term was often, but not exclusively, used as a synonym for severe agitation, as ‘acute disturbance’ and related phrases have been conventionally used in mental health settings. Use of the term ABD in mental health notes does not convey a diagnosis, only the perception of a person's presentation; however, the term's connection to a literature emphasising the high risk of physical health collapse and need for urgent treatment means its disproportionate use in Black people may contribute to existing racial inequalities in the use of coercive measures during crisis presentations. Indeed, psychiatrists may be inadvertently contributing to the labelling of Black people's behaviour as the ‘diagnosis’ ABD. Use of the term ABD, especially without reference to supporting clinical evidence, may be the result of the same underlying racist assumptions, conscious or unconscious, that have been implicated in causing the racial differences seen in coercive treatment.^10^

These findings support the suggestion made by the Royal College of Psychiatrists’ recent Position Statement^11^ that we should move away from using the term ABD and instead adopt a descriptive term, such as McGuinness and Lipsedge's suggested ‘severely agitated person in distress’,^8^ which does not imply a coherent underlying diagnostic entity and does not rely on racially loaded clinical descriptions. This would be a more meaningful description for the majority of patients described as experiencing ABD in this study, and distinguish them from the substantial minority where the term ABD was being used in the absence of severe agitation. There would also be benefit in creating a more rigorous descriptor for the much smaller group of patients who present to emergency services with highly disturbed behaviour who are in a physiologically compromised state that requires urgent physical healthcare. Using the label ABD for this group is inappropriate in framing the presentation in terms of behaviour without explicit reference to the physiological concerns, not least because of the far more common use of the term to describe agitated behaviour in all settings, not just in mental health. An alternative name that makes clear that physiological compromise is central to the diagnosis, and a set of clinical diagnostic features that focus on objective markers of physiological distress and remove subjective descriptors vulnerable to racialised bias such as ‘superhuman or excessive strength’, would, in our opinion aid, the detection of the small group at risk of life-threatening physiological compromise. However, we are mindful that changing terminology alone without challenging the underlying assumptions creating current inequalities will not be sufficient to eliminate them.

## Data Availability

C.P. and P.D. had access to all of the data used in this analysis. The data-sets generated and/or analysed during the current study are not publicly available. Data are owned by a third party, the Biomedical Research Centre Clinical Records Interactive Search tool, which provides access to anonymised data derived from the South London and Maudsley NHS Foundation electronic medical records. These data can only be accessed by permitted individuals with honorary contracts with the Trust from within a secure firewall (i.e. remote access is not possible and the data cannot be sent elsewhere), in the same manner as the authors.

## Supplementary material

For supplementary material accompanying this paper visit http://doi.org/10.1192/bjo.2023.528. click here to view supplementary material
