# Supplementary material for: Use of ‘acute behavioural disturbance’ in mental health records: differences over time and by ethnicity in a London NHS mental health Trust
Source: BJPsych Open. 2023 Jul 24;9(4):e133. doi: 10.1192/bjo.2023.528 (PMC10375904; doi:10.1192/bjo.2023.528)
Supplement: Supplementary file 1 [file S2056472423005288sup001.docx]

### Supplementary table 1: Counts and rates of ABD terms and current ABD by year, 2006-2021

| **Year** | **Documents (1000s)** | **ABD terms** | **Rate ABD terms/100,000** | **Current ABD** | **Rate Current ABD/100,000** |
| --- | --- | --- | --- | --- | --- |
| 2006 | 47.6 | 0 | 0 ( 0 - 7.74 ) | 0 | 0 ( 0 - 7.74 ) |
| 2007 | 105.3 | 1 | 0.95 ( 0.02 - 5.29 ) | 1 | 0.95 ( 0.02 - 5.29 ) |
| 2008 | 134.1 | 6 | 4.47 ( 1.64 - 9.74 ) | 5 | 3.73 ( 1.21 - 8.7 ) |
| 2009 | 142.1 | 5 | 3.52 ( 1.14 - 8.21 ) | 2 | 1.41 ( 0.17 - 5.09 ) |
| 2010 | 148.5 | 5 | 3.37 ( 1.09 - 7.86 ) | 3 | 2.02 ( 0.42 - 5.91 ) |
| 2011 | 149.6 | 12 | 8.02 ( 4.15 - 14.01 ) | 6 | 4.01 ( 1.47 - 8.73 ) |
| 2012 | 152.1 | 20 | 13.15 ( 8.03 - 20.31 ) | 12 | 7.89 ( 4.08 - 13.78 ) |
| 2013 | 164.8 | 9 | 5.46 ( 2.5 - 10.37 ) | 7 | 4.25 ( 1.71 - 8.75 ) |
| 2014 | 164.2 | 16 | 9.74 ( 5.57 - 15.82 ) | 7 | 4.26 ( 1.71 - 8.78 ) |
| 2015 | 164.8 | 21 | 12.74 ( 7.89 - 19.48 ) | 15 | 9.1 ( 5.09 - 15.01 ) |
| 2016 | 192.4 | 32 | 16.63 ( 11.38 - 23.48 ) | 17 | 8.84 ( 5.15 - 14.15 ) |
| 2017 | 213.4 | 30 | 14.06 ( 9.48 - 20.07 ) | 18 | 8.43 ( 5 - 13.33 ) |
| 2018 | 206.6 | 33 | 15.97 ( 11 - 22.43 ) | 15 | 7.26 ( 4.06 - 11.98 ) |
| 2019 | 210.5 | 23 | 10.92 ( 6.92 - 16.39 ) | 17 | 8.07 ( 4.7 - 12.93 ) |
| 2020 | 222.4 | 35 | 15.74 ( 10.96 - 21.89 ) | 28 | 12.59 ( 8.37 - 18.2 ) |
| 2021 | 214.5 | 70 | 32.63 ( 25.43 - 41.22 ) | 41 | 19.11 ( 13.71 - 25.92 ) |
